# Treatment with a Monoclonal Anti-IL-12p40 Antibody Induces Substantial Gut Microbiota Changes in an Experimental Colitis Model

**DOI:** 10.1155/2016/4953120

**Published:** 2016-01-06

**Authors:** Josué Castro-Mejía, Maja Jakesevic, Łukasz Krych, Dennis S. Nielsen, Lars H. Hansen, Bodil C. Sondergaard, Peter H. Kvist, Axel K. Hansen, Thomas L. Holm

**Affiliations:** ^1^Department of Food Science, Faculty of Science, University of Copenhagen, Rolighedsvej 26, 1958 Frederiksberg, Denmark; ^2^Department of Veterinary Disease Biology, Faculty of Health and Medical Science, University of Copenhagen, Thorvaldsensvej 57, 1870 Frederiksberg, Denmark; ^3^Department of Environmental Science, Aarhus University, Frederiksborgvej 399, 4000 Roskilde, Denmark; ^4^Novo Nordisk Park, 2760 Maaloev, Denmark

## Abstract

*Background and Aim*. Crohn's disease is associated with gut microbiota (GM) dysbiosis. Treatment with the anti-IL-12p40 monoclonal antibody (12p40-mAb) has therapeutic effect in Crohn's disease patients. This study addresses whether a 12p40-mAb treatment influences gut microbiota (GM) composition in mice with adoptive transfer colitis (AdTr-colitis).* Methods*. AdTr-colitis mice were treated with 12p40-mAb or rat-IgG2a or NaCl from days 21 to 47. Disease was monitored by changes in body weight, stool, endoscopic and histopathology scores, immunohistochemistry, and colonic cytokine/chemokine profiles. GM was characterized through DGGE and 16S rRNA gene-amplicon high-throughput sequencing.* Results*. Following 12p40-mAb treatment, most clinical and pathological parameters associated with colitis were either reduced or absent. GM was shifted towards a higher Firmicutes-to-Bacteroidetes ratio compared to rat-IgG2a treated mice. Significant correlations between 17 bacterial genera and biological markers were found. The relative abundances of the RF32 order (Alphaproteobacteria) and* Akkermansia muciniphila* were positively correlated with damaged histopathology and colonic inflammation.* Conclusions*. Shifts in GM distribution were observed with clinical response to 12p40-mAb treatment, whereas specific GM members correlated with colitis symptoms. Our study implicates that specific changes in GM may be connected with positive clinical outcomes and suggests preventing or correcting GM dysbiosis as a treatment goal in inflammatory bowel disease.

## 1. Introduction

Inflammatory bowel disease (IBD) arises from a loss of tolerance and excessive immune response to commensal bacteria in a genetically susceptible host, although environmental factors may influence as well. Ulcerative colitis (UC) and Crohn's disease (CD) are the major forms of IBD affecting the gastrointestinal tract [1]. Key clinical features of these diseases include abdominal pain, weight loss, and diarrhea, which can be hemorrhagic [1–3]. As many as 1.4 million in the United States and 2.2 million persons in Europe suffer from IBD and the numbers are increasing [4].

Animal models are widely used to study and explain the pathological mechanisms of gut inflammation. In order to study IBD and evaluate anti-inflammatory strategies, a variety of animal models have been developed and are traditionally divided into those with spontaneous development of colitis due to genetic manipulation (e.g., targeted deletion of the anti-inflammatory cytokine IL-10), chemically induced colitis (e.g., dextran sulfate sodium (DSS)), hapten-induced colitis (e.g., 2,4,6-trinitrobenzene sulfonic acid (TNBS)), and adoptive transfer (AdTr) models (transfer of T cells from a donor mouse to a T cell deficient mouse) [5]. Compared to chemically inducible models of colitis such as those based upon DSS and TNBS, AdTr of CD4^+^CD25^−^ T cells into immune deficient mice more closely reflects the altered gene expression in human IBD [6]. Mouse models of colitis induced by AdTr of CD25^−^ depleted CD4^+^ T cells are highly suitable for pharmacological testing of new IBD drug candidates because they are easy and fast to perform (compared to other chronic models, e.g., SAMP-1/Yit) [7, 8], there is no generation of anti-drug antibodies, and it results in uniform and highly reproducible clinical and pathological signs of colitis [9]. Three weeks after adoptive cell transfer most recipient mice develop clear signs of colitis, characterized by weight loss, loose stools, increased white blood cell (WBC) count, and thickened and shortened colon [5].

Anti-IL-12/23p40 monoclonal antibody (mAb) targets the p40 subunit common to IL-12. IL-12 is formed by two chains, IL-12p35 and IL-12p40, which together form the active heteromer IL-12p70 [10], whereas the active heterodimer IL-23 is formed by IL-12p40 and IL-12p19. Both IL-12 and IL-23 are produced by monocytes, macrophages, and dendritic cells in response to microbial stimulation [10]. IL-12 induces the generation of T helper cells type 1 (T_H_1) [11], and it enhances the cytotoxic activities of natural killer (NK) cells [12] leading to secretion of several cytokines, especially interferon-gamma (IFN-*γ*) [11]. IL-23 stimulates CD4^+^ T cells to differentiate into a novel subset of T helper cells called T_H_17 cells. T_H_17 cells produce proinflammatory cytokines that enhance T cell priming and stimulate the production of other proinflammatory molecules such as IL-1, IL-6, and TNF-*α*, as well as chemokines resulting in inflammation [13–15]. CD has been associated with excess cytokine activity mediated by T_H_1 and T_H_17 cells. IL-12 and IL-23 are increased in patients with CD, but their production is downregulated after administration of IL-12/23p40 monoclonal antibody [7, 13, 16, 17]. Clinical trials with anti-IL12/23p40 therapy have shown encouraging results in CD patients not responding to the first-line biologic treatment, which is anti-TNF-*α* mAb. The reason why anti-IL12/23p40 works particularly well in this patient segment is currently unknown, but it could be due to specific genetic alterations, as well as microbiota/gut interactions common in the IL-12/23p40 pathway driving the pathogenesis in these patients. Moreover, alterations in gut microbiota (GM) have been shown as a predictor of relapse in CD patients [18].

Genetically engineered animal models of IBD do not develop fulminant colitis under germ-free conditions, but gut inflammation evolves when they are colonized by bacteria, which points out the important role of the GM in the initiation and development of colitis [19, 20]. Moreover, antibiotics may ameliorate experimental colitis even in a therapeutic setting [7]. Several studies have shown that IBD is accompanied by a shift in GM towards higher abundance of proinflammatory bacteria, such as Enterobacteriaceae, while the abundance of, for example, lactobacilli and bifidobacteria is reduced [21–24]. Furthermore, a number of studies indicate that GM diversity is reduced in colitis [21, 25, 26]. However, detailed knowledge on how GM composition changes in relation to colitis is still limited and more studies are required to verify the alternations in the overall composition of GM during intestinal inflammation and to which extent such alternations are of importance in a preventive or therapeutic intervention. Identifying relevant links between GM composition and clinical parameters of colitis is also of great importance.

Consequently, the aim of the present study was to evaluate how microbiota composition in colonic content correlated with clinical signs of IBD in mice treated with monoclonal anti-IL12/23p40 antibody (12p40-mAb) in an adoptive T cell transfer model of colitis (AdTr-colitis).

## 2. Materials and Methods

### 2.1. Mice

All experiments were conducted in accordance with the European Communities Council Directive 86/609/ECC for the protection of animals used for experimental purposes, approved by the Danish Animal Experiments Inspectorate, Ministry of Food, Fisheries and Agriculture, Denmark, and the internal Ethical Review Council at Novo Nordisk A/S.

C.B-Igh-1b/IcrTac-Prkdcscid (C.B-17 SCID) and BALB/cAnNTac female mice (8–10 weeks) bred under barrier protected conditions (Taconic, Ll. Skensved, Denmark) were housed at Novo Nordisk A/S. Mice were identified using Plexx microchips (Plexx, Elst, Netherlands) and were randomized in the cages to reduce cage effects. Dirty cage bedding was transferred to the individual cages before the experiment was initiated as well as once weekly during the experiment. Health monitoring was performed according to FELASA guidelines [27].

### 2.2. Purification of Cells and Induction of Colitis

Colitis was induced by AdTr of CD4^+^CD25^−^ T cells (AdTr-colitis) from spleen of MHC-compatible BALB/c mice to C.B-17 SCID recipients as previously described [9]. Briefly, splenocytes of BALB/c donor mice were subjected for positive selection of CD4^+^ T cells using Dynabeads and DETACHaBEAD (Life Technologies Europe, Ballerup, Denmark) and depletion of CD25^+^ from the CD4^+^ T cell suspensions using the CD25 MicroBead kit. Flow cytometry was used to analyze purity of the cells and showed that more than 98% of the CD4^+^ cells were CD25^−^ cells. Each recipient was reconstituted with 300,000 cells by intraperitoneal injection. Two or three weeks after transfer, peripheral blood from all mice was analyzed by flow cytometry for the presence of CD4^+^ T cells. Only animals with successful transplantation of cells were included in the study.

### 2.3. Experimental Groups

The mice were divided into four groups. One group of nontreated C.B-17 SCID mice (SCID control) were not subjected to AdTr-colitis (*n* = 4), while the three groups of adoptively transferred C.B-17 SCID mice were treated with a neutralizing rat anti-mouse IL-12/23p40 monoclonal antibody (12p40-mAb) (clone C17.8) (*n* = 15) or rat-IgG2a monoclonal isotype antibody (clone 2A3) (*n* = 13) or NaCl (*n* = 14) from day 21 until termination at day 47. The antibodies were purchased from Bio X Cell (New Hampshire, USA) and had been tested for endotoxins. The antibodies were injected intraperitoneally three times weekly and dosed according to body weight at 25 mg kg^−1^.

### 2.4. Monitoring of Disease

Animals were weighed three times per week and mice that lost more than 20% of the initial weight were sacrificed. Fecal samples were evaluated and scored from 0 to 4 according to their consistency (normal stool = 0; slightly soft stool = 1; soft but formed stool = 2; not formed stool = 3; liquid stools or no feces in the colon at sacrifice = 4). Disease activity index score (DAI) was calculated based on data obtained from weight loss and feces type as previously described by Murthy et al. [28]. As fecal blood is rarely observed in AdTr-colitis it was not monitored. Mice were anaesthetized by isoflurane (Isoba Vet, MSD Animal Health, Ballerup, Denmark) and blood was obtained by retroorbital puncture. All blood samples were collected in EDTA K2 coated microtubes (Milian, Gahanna, Ohio, USA). EDTA-stabilized peripheral whole blood samples (20 *μ*L) were used for monitoring the number of white blood cells (WBC) per liter with Medonic CA 620 (Boule Nordic, Denmark) blood analysis apparatus according to the manufacturer's protocol. Colonoscopy was performed on days 21 and 34. Mice were anaesthetized with isoflurane and placed in dorsal recumbency. A rigid telescope (HOPKINS Straight Forward, 0°) was connected to a light source/air pump (Xenon 175) and camera (Telecam SL) as described by Becker et al. [29] The endoscope (2 mm) was coated with a lubricant containing lidocaine hydrochloride (Farco-Pharma, Köln, Germany) and introduced via the anus into the distal 4 cm of the colon. The evaluation of the colonoscopic findings was done by two blinded observers using the murine endoscopic index of colitis severity (MEICS) score (Supplemental Table  1 in Supplementary Material available online at http://dx.doi.org/10.1155/2016/4953120), as outlined by Becker et al. [30].

### 2.5. Postmortem Analysis

Mice were sacrificed by cervical dislocation on day 47. However, one mouse in the rat-IgG2a group was sacrificed at day 45 due to severe weight loss. After the animals were sacrificed the colon was excised and opened longitudinally. Fecal and colonic content was collected in a sterile Eppendorf tube and tissue was gently rinsed with saline and its weight (*W*, mg) and length (*L*, cm) were measured. The left halves of colon were used for cytokine measurements and the right halves were fixed on a plastic plate with pins and processed for histological analysis. Cecum was excised and its contents were collected in sterile Eppendorf tubes.

### 2.6. Histology

Tissue for histology was fixed in 4% paraformaldehyde (VWR-Bie & Berntsen, Herlev, Denmark) for approximately 24 hours at 4°C. Subsequently, the samples were transferred to 70% ethanol and stored at 4°C until processed for histopathology. The samples were processed in a Leica Asp300S histoprocessor (Leica Microsystems, Ballerup, Denmark) overnight, embedded in paraffin blocks using a Shandon Histocentre 3 (Thermo Electron Corporation, Marietta, Ohio), and sectioned at a thickness of 3 *μ*m in a Leica Microtome RM 2165 (Leica Microsystems, Ballerup, Denmark). Subsequently, the slides were stained with hematoxylin (Ampliqon, Skovlunde, Denmark) and eosin (Sigma-Aldrich, Brøndby, Denmark) (H&E) for light-microscopic examination, Olympus Ax70 microscope. The severity of the histopathological lesions of colon segments was examined in a blinded manner, using the criteria described in Supplemental Table  2.

For immunohistochemical (IHC) detection of CD3 or S100A8 (calprotectin) paraformaldehyde fixed colon sections were mounted on adhesive slides (Superfrost Plus, Menzel-Gläser, Germany) dried at 60°C and kept at 4°C until processed. Tissue sections were processed through xylene and rehydrated. For CD3 IHC the tissue sections were boiled in Tris-EGTA buffer and quenched with H_2_O_2_ (0,5%). Tissue sections were blocked (with goat and mouse serum, BSA, and skim milk) and slides were incubated with polyclonal rabbit anti-human CD3 antibody (RM-9107-S; SP7, Thermo Scientific). After washing in TBS buffer secondary antibody-polymer complex (Envision, K4003) was applied. Then, slides were developed with diaminobenzidine (DAB) and counterstained in Meyer's hematoxylin and mounted with Pertex.

For calprotectin IHC staining the tissue sections were boiled in citrate buffer and quenched with H_2_O_2_ (0,5%). Endogenous biotin was blocked using an avidin-biotin blocking kit. Nonspecific binding was blocked by incubation with TBS containing skimmed milk, donkey serum, and mouse serum. The primary antibody (Rat-a-Mo S100A8 (MRP-8), MyBiosource) and secondary antibody (Biotin-SP Donkey Anti-Rat IgG, Jackson ImmunoResearch) were diluted in Tris-buffer containing skimmed milk and donkey and mouse sera and incubated for 60 min each at room temperature. Next, an amplification step was performed by incubation with Vectastain ABC Peroxidase Kit for 30 min followed by a chromogenic reaction with DAB. Nuclei were counterstained with Meyer's hematoxylin and the sections were rehydrated, cleared in xylene, and mounted with Pertex. Control immunostainings were run without the primary antibody and with a nonsense polyclonal antibody of the same concentration as the primary antibody for both CD3 and calprotectin.

Automated digital image analyses were performed on the IHC positive areas using the Visiopharm Integrator System (VIS, version 4.5.1.324, Visiopharm, Hørsholm, Denmark). On individual digital images of the proximal and distal colon sections, a region-of-interest (ROI) was automatically defined of both colon sections using *K*-means clustering classification. Subsequently, an analysis was run using threshold classification inside the ROI to detect the brown DAB staining of the specific calprotectin IHC immunostaining. The results are given as area stained with CD3 or calprotectin of the entire colon sections (%).

### 2.7. Cytokine Measurements

Tissue for cytokine analysis was weighed and transferred to individual 3.6 mL CryoTubes containing Tissue Homogenate Lysis Buffer (Ampliqon, Skovlunde, Denmark). The buffer was a solution of 200 mM NaCl, 5 mM EDTA, 10 mM Tris, 10% glycerin, 1 mM PMSF, 1 *μ*g mL^−1^ leupeptin, and 28 *μ*g mL^−1^ aprotinin (pH 7.4). The buffer was kept cold (approx. 4°C) at all times. The tubes were immediately snap-frozen in liquid nitrogen and stored at −80°C until homogenized. Using an Ultra-Turrax T25 basic disperser (IKA-Werke, Staufen, Germany) the colon segments were homogenized and the homogenates were centrifuged three times for 15 min at 10,000 ×g and 4°C, twice in Eppendorf tubes and the last time in an Ultrafree MC-Centrifugal Filter device, 5 *μ*m pore size (Millipore, Billerica, Massachusetts, USA). The supernatants were analyzed for levels of colonic cytokines and chemokine using Milliplex (Millipore, Billerica, Massachusetts, USA). The assays were run according to the manufacturer's guidelines with the exception that the standard and test samples were diluted in the Tissue Homogenate Lysis Buffer rather than the kit-supplied assay diluents.

### 2.8. Gut Microbiota Characterization

Cecal, colonic, and fecal contents collected at euthanasia were homogenized three times for 20 sec at speed 5.5 m/sec prior to extraction using a Bead Beater (FastPrep-24, MP Biomedicals, Santa Ana, USA). DNA was extracted using the QIAamp DNA Stool Mini Kit (Qiagen, Hilden, Germany) according to the manufacturer's instructions. Concentration and quality of the extracted DNA were measured using a NanoDrop 1000 Spectrophotometer (Thermo Scientific, USA). A preliminary screening of the GM diversity (based on profiles of 16S rRNA amplicons) of cecal, colonic, and fecal contents was performed using denaturing gradient gel electrophoresis (DGGE) as outlined in Pyndt Jørgensen et al. [31]. Only DNA extracted from colon contents was subjected to high-throughput sequencing (Illumina MiSeq). The V3-V4 region of the 16S rRNA gene (amplicon size ~460 bp) was amplified with primers including adapters for the Nextera Index Kit (Illumina, California, USA). The primers sequences, conditions for amplification and tagmentation (1st and 2nd round of PCR), purification, and sequencing were performed as previously described [31]. Pair-ended reads (with corresponding quality scores) were trimmed and merged using the CLC Genomic Workbench 7.0.4 (CLC bio, Aarhus, Denmark) [31]. The UPARSE algorithm [32] was used for OTU clustering (97%) and filtering of chimeric sequences, while the GreenGenes database 97% (version 12.10) was used for OTU picking [33]. The dataset was further analyzed using the Quantitative Insight Into Microbial Ecology (QIIME versions 1.7.0 and 1.8.0) [34].

Relative quantification of* Akkermansia muciniphila* was carried out as previously described [35]. Briefly, the reaction mixture (20 *μ*L) contained 10 *μ*L of 1x SYBR green PCR Master Mix (Applied Biosystems), 1 *μ*L of either* A. muciniphila* specific-primers or 16S rRNA universal primers (each primer was used at a final concentration 0.5 *μ*M), 4 *μ*L of nuclease-free water, and 5 *μ*L of DNA template (20 ng *μ*L^−1^). The temperature profile for qPCR was as follows: 95°C for 5 min and 40 cycles of 95°C for 15 s and 60°C for 1 min. For generation of the standard curve, serial tenfold dilutions of* A. muciniphila* DSMZ 22959 genomic DNA were used.

### 2.9. Statistical Analyses

General statistical analyses were carried out using GraphPad Prism version 5.01 (GraphPad Software, La Jolla, California, USA). Unpaired Student's *t*-test was used when comparing the means of normally distributed parametric data from two groups and a Mann-Whitney *U* test was performed when comparing non-Gaussian distributed data from two groups. When three or more groups with parametric data were compared one-way ANOVA was used with Tukey's posttest. The Kruskal-Wallis with Dunn's posttest was used on multiple datasets that did not assume Gaussian distributions. For high-throughput sequencing, the number of sequences used for subsample (−*e* value, Alpha and Beta Diversity; −*d* value, multiple rarefactions) was set to 90% of the most indigent sample. The Jackknife Beta Diversity workflow was used to generate PCoA plots (based on 10 distance matrices that were determined using 10 subsampled OTU tables). Differences between categories in UniFrac distance matrices were assessed with analysis of similarities (ANOSIM). Alpha Diversity was measured and expressed as observed species (97% similarity OTUs) and computed with 10 rarefied OTU tables. Comparison of Alpha Diversities was made through nonparametric *t*-test method (Monte Carlo, 999 permutations). The occurrence and absence of OTUs associated with a given group of mice were assessed with the *G*-test of independence, whereas differences in the abundance of OTUs were tested with ANOVA. Finally, differences in the relative abundance of* A. muciniphila* determined by qPCR were evaluated using two-tailed Student's *t*-test.

## 3. Results

### 3.1. Monitoring of Disease (DAI Score, Weight Change, and Pathology)

**Figure 1:**
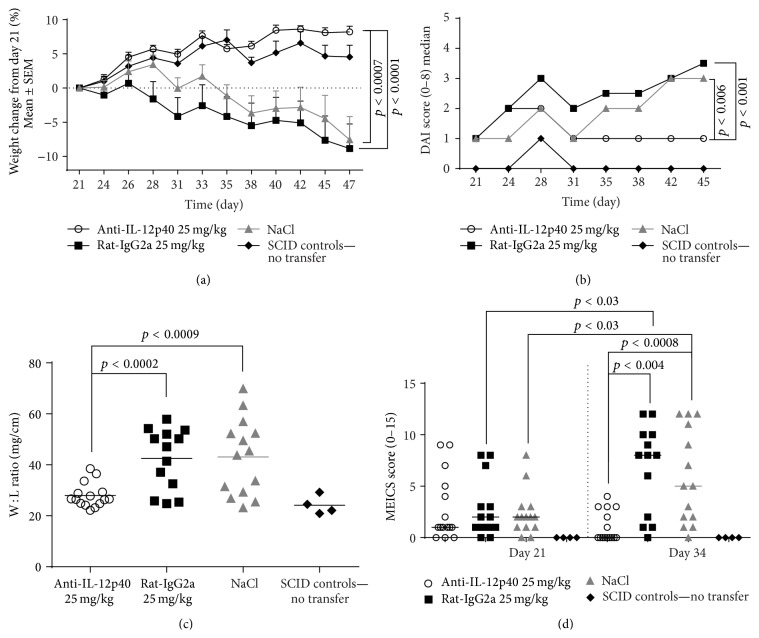
Induction of AdTr-colitis is associated with weight loss, loose stools, and colonic inflammation. (a) Delta weight loss from day 21 is shown as mean ± SEM for individual groups. Significant differences between the areas under the curve (AUC) for individual mice were determined by Student's *t*-test. (b) DAI was determined with a combined scale from 0 to 8 by combining weight loss (0–4) and stool score (0–4). Differences in DAI scores were calculated as AUC for individual mice from 21 to 45 days using Mann-Whitney *U* test. (c) The colon weight- (mg) to-length (cm) ratio was calculated for individual mice. Comparisons between groups were performed using Student's *t*-test. (d) Endoscopic analysis of colon at 21 and 34 days was graded with MEICS. All mice were scored semiquantitatively in a blinded fashion. Significant differences were determined with Mann-Whitney *U* test for unpaired analysis and Wilcoxon for paired analysis.

The CD4^+^CD25^−^ T cell transfer was well tolerated by the mice, and no adverse events directly related to 12p40-mAb, rat-IgG2a isotype control, or NaCl were detected. Mice were randomized according to body weight (BW) on day 19. No apparent signs of disease were observed within the first two weeks after AdTr-colitis (data not shown). The change in BW over the course of the experiment from days 21 to 47 is shown in Figure 1(a). Mice started to lose weight in the 3rd-4th week following transfer. The weight loss progressed until the termination of the study. Mice treated with 12p40-mAb did not show any apparent weight loss and were similar to SCID control mice, whereas mice treated with the isotype control and NaCl developed severe weight loss (*p* < 0.0001 and *p* < 0.0007). No significant differences were observed between NaCl and rat-IgG2a treated groups. The disease activity index (DAI) is a combined index from 0 to 8 containing weight loss (score 0–4) and stool score (score 0–4), which gives an overall evaluation of the disease intensity during the study. DAI was significantly lower in mice treated with 12p40-mAb compared to rat-IgG2a (*p* < 0.001) and NaCl (*p* < 0.006) (Figure 1(b)). In animal models of AdTr-colitis, disease can be associated with thickening and shortening of the colon wall as a host inflammatory response. As expected, the colon *W* : *L* ratio was increased in mice receiving NaCl and rat-IgG2a. A significant treatment effect was observed with the 12p40-mAb compared to the isotype treated group (*p* < 0.0002) and NaCl (*p* < 0.0009) (Figure 1(c)), whereas no differences were observed between their controls groups.

Endoscopic pictures were acquired for monitoring and grading inflammation using MEICS (encompassing the thickening of the colon, changes of vascular pattern, visible fibrin, granularity of mucosal surface, and stool consistency). After randomization but prior to treatment (day 21), the majority of mice had clear signs of colonic inflammation; nevertheless, no significant difference between the groups could be detected (Figure 1(d)). At day 34, however, mice treated with the 12p40-mAb had a significant reduction in the endoscopic score, compared to mice treated with rat-IgG2a (*p* < 0.004) and NaCl (*p* < 0.0008). No differences were observed between the 12p40-mAb treated and healthy SCID control mice. Similarly, no difference was observed between the rat-IgG2a and NaCl treated mice at day 34 (Figure 1(d)). When comparing disease progressions within the groups at day 21 versus day 34, mice treated with rat-IgG2a and NaCl increased their endoscopic score, from day 21 to day 34 (both *p* < 0.03). In comparison, mice treated with the IL12p40-mAb showed signs of disease amelioration, *p* < 0.09 (Figure 1(d)).

### 3.2. Histopathology and Immunohistochemical Staining

**Figure 2:**
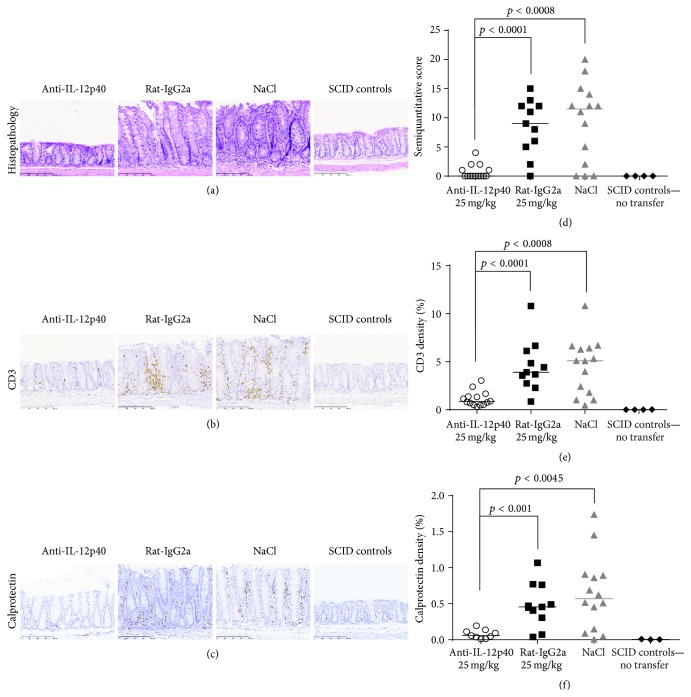
Histology and immunohistochemistry of representative areas of mouse colon. The left panel (a, b, c) shows the histopathological and IHC evaluation of the colon. After H&E staining (a), the 12p40-mAb treated mice had normal morphology as seen in the SCID control mice. Rat-IgG2a and NaCl treated mice had major hyperplasia of the mucosal layer and infiltration of immune cells. The CD3 (b) and calprotectin (c) IHC identify these immune cells as being CD3 or calprotectin positive cells as visualized with the brown DAB staining of the positive cells in the mucosa layer and submucosa. The scale bar indicates 200 *μ*m and all images are captured at 20x magnification. The images are from the mouse with the results representing the group median for the specific staining of the four mice groups. The right panel (d, e, f) depicts the results from the histopathological (d) evaluation using a semiquantitative score (range 0–24). CD3 (e) and calprotectin (f) staining were quantitatively assessed by digital image analysis; data is represented as the density of the IHC reactivity (%).

At day 47, a significant reduction in histopathology scores was observed in mice treated with 12p40-mAb compared to the corresponding isotype control (*p* < 0.0001) and NaCl (*p* < 0.0008) (Figures 2(a) and 2(d)), while no significant differences were observed between the control groups. Since pathogenic CD4 T cells drive disease progression in AdTr-colitis, colonic sections were stained for a T cell marker (CD3). The percentage of CD3 stained colon tissue was then used to monitor the effect of 12p40-mAb on colonic T cell inflammation. Mice treated with 12p40-mAb had significantly lower density of CD3 as compared to the isotype control and NaCl (*p* < 0.0001 and *p* < 0.0008, resp.) (Figures 2(b) and 2(e)). Calprotectin is primarily found in the cytosol of neutrophils and is dramatically upregulated in areas of inflammation, whereas it is seldom observed in healthy tissue. Compared to the isotype control and NaCl treatments, mice treated with 12p40-mAb had lower density of calprotectin (or neutrophil inflammation) in the colon (*p* < 0.001 and *p* < 0.0045, resp.) (Figures 2(c) and 2(f)). No significant differences were determined between the control groups.

### 3.3. Cytokines

Total colonic cytokine/chemokine levels were determined in colon biopsies from mice treated with 12p40-mAb or rat-IgG2a or healthy controls. Several proinflammatory cytokines and chemokines (TNF-*α*, CCL5, IP-10, and KC) were significantly elevated in mice treated with rat-IgG2a compared to 12p40-mAb treated mice (Table 1). The cytokine/chemokine levels in 12p40-mAb treated mice were not different from the healthy control mice (Table 1). Moreover, mice treated with 12p40-mAb had significantly higher levels of T_H_2 (IL-5, IL-9, and IL-13) and the immunosuppressive cytokine IL-10 compared to rat-IgG2a treated mice (Table 1). Thus, different T cell environments may be induced by the two treatment settings.

### 3.4. Gut Microbiota Composition

**Figure 3:**
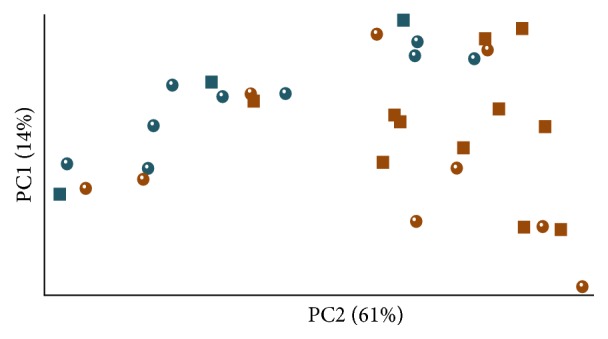
Beta Diversity analysis separates nonactive disease [light-blue] (12p40-mAb [circles] and healthy [squares]) from active colitis mice [orange] (rat-IgG2a [circles] and NaCl [squares]). PCoA plot (unscaled) was determined through weighted UniFrac metrics (ANOSIM, *p* < 0.01, *r* = 0.18) of the colonic microbiota from 32 mice. The ellipsoids highlight the degree of variation around each sample.

Significant changes of 16S rRNA amplicons profiles, based on denaturing gradient gel electrophoresis (DGGE), were observed within gut contents of the NaCl and 12p40-mAb treated mice (Supplemental Figure  1). Since significant differences between the colon contents of 12p40-mAb and rat-IgG2a were also observed, the DNA from colonic contents was subjected to high-throughput sequencing (Illumina MiSeq). The number of reads obtained by sequencing of 16S rRNA gene (V3-V4 region) amplicons from 32 colonic fecal samples was 1,697,168 (mean sequence length of 341 bp). After preprocessing (trimming, quality control, sorting, and chimera filtering), the number of high quality reads obtained was 990,505, whereas the number of sequences per sample varied from 14,534 to 61,423 (average of 30,953 ± 9,561). The estimated average number of observed OTU-species in the 12p40-mAb treated group (*n* = 9), SCID control (*n* = 3), rat-IgG2a treated group (*n* = 9), and NaCl group (*n* = 11) was not significantly different [a reduced number of samples were subjected to sequencing due to limited sequencing capacity; samples were randomly selected and all treatments were represented] (*t*-test, *p* > 0.05; Supplemental Table  3). Similarly, no differences in Beta Diversity were observed between the 12p40-mAb and SCID control (nonactive disease) or between the isotype and NaCl treated mice (active colitis) (ANOSIM, *p* > 0.05). Principal coordinate analysis of weighted UniFrac distance matrices, however, showed significant separation of the nonactive disease and active colitis mice (ANOSIM, *p* < 0.01, weighted *r* = 0.18; Figure 3). Furthermore, their GM showed changes in the relative abundance of 7 genera (Table 2) [not statistically different after correcting for multiple comparisons] and at the phylum level a significantly lower (*t*-test, *p* > 0.05) Firmicutes : Bacteroidetes ratio was seen in the colitis active mice (0.30) as compared to the nonactive disease mice (0.83) (however, when only 12p40-mAb and rat-IgG2a were compared no significant differences were observed, putatively due to low number of observations and high intersample variation). Such lower relative abundance of operational taxonomic units (OTUs) assigned to phylum Firmicutes was partially caused by a drop in the relative abundance of Clostridiales from 34.5% to 15.9% (constituted by members of* Ruminococcus*,* Oscillospira*, and an unclassified genus).

### 3.5. Correlation of Gut Microbiota Composition to Disease Parameters

**Figure 4:**
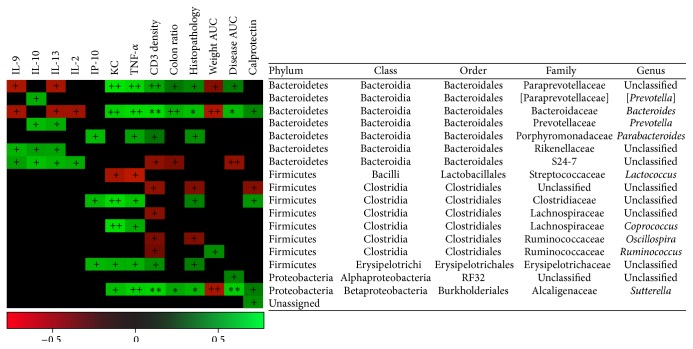
Pearson's correlation of immunological and host parameters with changes in the relative distribution of microbiota members of the colonic content. On top of the heat map the immunological and host parameters are depicted. The lineages are marked on the right and are represented by five major taxonomic ranks. Symbols describing significant correlations were defined as follows: for *p* value, + ≤ 0.05 and ++ ≤ 0.01. For *q*-value (False Discovery Rate correction), *∗* ≤ 0.05 and *∗∗* ≤ 0.01. A matrix containing the *r*-values for every significant correlation is shown in Supplemental Table  4.

Seventeen genus-level OTUs correlated either positively or negatively with changes in host parameters (Figure 4 and Supplemental Table  4). Within highly abundant members,* Bacteroides* (with a range in relative distribution of 1.5–89%) showed strong positive correlations with most host parameters (with exception of weight AUC) as well as inverse correlations against IL-2, IL-9, and IL-13. Two unclassified genera of Clostridiales (1.2–63%, unclassified Clostridiales and unclassified Lachnospiraceae),* Oscillospira* (0.1–8.5%), and S24-7 family (3–60%) correlated only negatively with the host parameters, while the latter group correlated positively with IL-2, IL-9, IL-10, and IL-13 (Figure 4). The rest of the OTUs that showed significant correlations did not exceed 15% of relative distribution.

**Figure 5:**
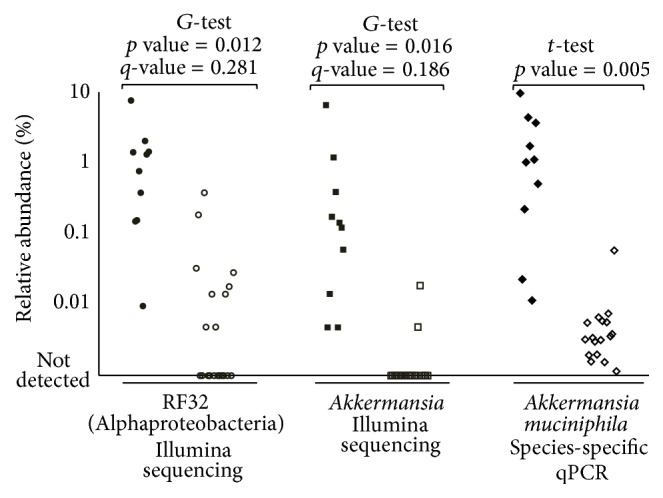
Bacterial prevalence in mice with high and low histopathology scores. Filled marks illustrate scores between 6 and 24, while empty marks illustrate those between 0 and 2. Samples in which RF32 (Alphaproteobacteria),* Akkermansia* (Illumina sequencing), and* A. muciniphila* (species-specific qPCR) were detected are represented by their relative distribution. Samples that were negative (no bacteria detected) are depicted at the no detection line. *q*-values were determined with the False Discovery Rate correction method.

Upon categorization of host parameters (intervals scores) the prevalence of two OTUs at the genus-level, assigned to* Akkermansia* (relative abundance of 0–7.8% and ≤0.001–11.6% as determined by Illumina sequencing and species-specific qPCR, resp.) and an unclassified member of the RF32 order (0.1 to 9.2%, Alphaproteobacteria related to* Rhodospirillum rubrum*), was associated (*G*-test, *p* < 0.05) with high histopathology scores (Figure 5). Furthermore, species-specific qPCR confirmed that an increase in relative abundance of* Akkermansia muciniphila* was associated with high scores of the same host parameter (Figure 5). Likewise, the prevalence of both bacteria was significantly associated also with high scores of CD3 density (scores > 2.7), disease AUC (scores > 39.5), and calprotectin (scores > 0.15). Additionally, prevalence of RF32 order was also observed in high scores of colon ratio (>33.7).

## 4. Discussion

Several studies support a causal role of a dysfunctional mucosal barrier in the manifestation of IBD. Recent findings have shed light on the mechanisms by which intestinal epithelial cells, microbiota, and immune cells interact and react in such an environment and how loss of normal regulatory processes may lead to IBD [17–20]. We have focused on the regulation of GM following treatment with a monoclonal antibody against IL-12p40 in an AdTr-colitis mouse model, in which IBD development was clearly prevented by the treatment. Most clinical and pathological signs in treated mice were either clearly reduced or not apparent at all. In other words, mice subjected to both AdTr-colitis and 12p40-mAb treatments appeared similar to SCID control mice, whereas mice treated with the isotype antibody developed severe signs of IBD. Comparable results have been achieved by others in the DSS model [36]. Changes in GM seem to be a shared primary phenomenon in both IBD patients and AdTr-colitis mice, and our results show that treatment with 12p40-mAb restores the GM composition, that is, similar to SCID mice. In addition, the colonic cytokine response with increased levels of IL-5, IL-9, IL-10, and IL-13 clearly shows that it induces a T_H_2 response, which is expectable when IL-12/IL-23 is prevented from inducing a T_H_1/T_H_17 profile. In contrast to what has been observed in CD patients [37] and the mice treated with the isotype antibody, rodents subjected to the 12p40-mAb treatment had a higher ratio of Firmicutes : Bacteroidetes. Furthermore, in CD patients a reduction in bacterial diversity as well as lower relative distribution of bacteria with anti-inflammatory properties has been reported [18, 38]. In our study, however, the overall diversity of the GM was not changed by the treatment. Whether dysbiosis or even specific bacterial strains can be future surrogate markers for inflammation, incomplete disease control, relapse, and response remains uncertain. However, caution should be taken when extrapolating from mouse to human. On the other hand, the increased Firmicutes : Bacteroidetes ratio in mice treated with 12p40-mAb was mainly due to the fact that the treated mice had higher proportion of Clostridiales members, many of which correlated inversely to clinical symptoms and TNF-*α* levels.* Clostridium* spp. are strong inducers of Foxp3-positive regulatory T cells in the colonic mucosa of mice [39], and transfer of a mixture of 17 human* Clostridium* spp. or related bacteria to mice also increased their level of regulatory T cells [40]. This fits nicely with our observation that 12p40-mAb significantly upregulated IL-10 in colon. In our study, an unclassified genus of Clostridiaceae family correlated positively to clinical symptoms, presence of effector cells and proinflammatory cytokines, but this is not necessarily in conflict with the other observations, as there are also a number of Clostridiales families in mice, which are known to induce inflammation [41].

Mice treated with 12p40-mAb had lower relative abundance of* Bacteroides*, and we observed a positive correlation between the abundance of* Bacteroides* with clinical symptoms and TNF-*α* levels. This is in accordance with the fact that both* B. vulgatus* [42] and* B. fragilis* [43] are known to induce intestinal inflammation in rodent models. It is of interest to note that the abundance of* Prevotella* correlates positively with IL-10 levels. In the DSS model they are known to increase severity of IBD [44, 45], but it is difficult to compare the DSS model with the AdTr model in this aspect as only few of the IL-10 producing cells are present in the recipient SCID background, and thus, their IL-10 levels will be different at baseline. It is well known that shifts in GM may lead to a change in the T_H_1/T_H_2/T_H_17 balance, which again may prevent symptoms of T_H_1/T_H_17 induced diseases such as CD. It, however, seems to be novel that an anti-T_H_1/T_H_17 treatment, such as 12p40-mAb, also leads GM towards an anti-inflammatory direction. In our controlled setup, correlations between mice GM members and biological markers were significantly associated, but this is seldom the case in humans [18]. On the other hand some mechanisms seem to be shared despite this lack of heterogeneity.

Surprisingly* A. muciniphila* correlated with high expression of clinical IBD symptoms as determined by histopathology.* A. muciniphila* is a mucin-degrader with high mucinase activity [46], which accounts for more than 1% of the bacterial cells in human feces [47]. Extracellular vesicles of* A. muciniphila* protect against DSS induced IBD in mice [48], and its prevalence is decreased manyfold in patients with either UC or CD [49]. Nevertheless, one possible explanation of our results is that intestinal inflammation may induce higher production of mucin that would primarily be degraded by* A. muciniphila* [50]. In the azoxymethane/DSS model of colon cancer* A. muciniphila* also seems to correlate to an increased number of colon tumors [51]. The prevalence of Alphaproteobacteria was found to be associated with high histopathological scores, they have previously been reported to be highly prevalent in fecal microbiota of UC patients [52], and their role in IBD pathogenesis seems to be associated with proinflammatory changes and GM dysbiosis [53].

## 5. Conclusions

Here we report that 12p40-mAb treatment in an AdTr-colitis mouse model of colitis leads to GM changes and that specific GM composition and members correlate with histopathological changes and cytokine responses. In addition, increasing the proportion of a number of Clostridiales members seems to be associated with prevention and attenuation of colitis symptoms. Thus, our results encourage the search for biomarkers based on GM and prevention or correction of dysbiosis as a potential treatment in IBD.

## Supplementary Material

Supplemental Material includes a Supplemental Figure and 4 Supplemental Tables. The Supplemental Figure contains DGGE profiles of 16S rRNA gene-amplicon of fecal, colon and cecum samples. The Supplemental Tables contain Murine endoscopic index of colitis severity (Supplemental Table 1), criteria for assignment of histopathology damage (Supplemental Table 2), bacterial richness as the number of OTU-species observed in colonic content (Supplemental Table 3) and r-values for significant correlations of immunological and host parameters with GM members (Supplemental Table 4).

## Figures and Tables

**Table 1:** Cytokine and chemokine levels in colon.

pg/100 mg colon^A^	Mice group			Significance	Significance
	SCID healthy control	12p40-mAb	rat-IgG2a	12p40-mAb versus rat-IgG2a	12p40-mAb versus SCID
CCL5	192 ± 72	229 ± 35	493 ± 62	<0.001	0.600
IL-2	182 ± 24	156 ± 19	95 ± 11	0.010	0.430
IL-5	69 ± 15	118 ± 37	31 ± 3	0.020	0.400
IL-9	1,489 ± 180	1,379 ± 194	683 ± 97	0.004	0.730
IL-10	164 ± 30	156 ± 22	91 ± 10	0.010	0.840
IL-13	932 ± 145	763 ± 122	392 ± 52	0.009	0.430
IP-10	926 ± 567	965 ± 180	3,446 ± 606	<0.001	0.930
KC	224 ± 44	167.5 ± 31	440 ± 61	<0.001	0.310
TNF-*α*	28 ± 5	24 ± 5	60 ± 7	<0.001	0.630

^A^Cytokine and chemokine levels in colon were evaluated by Luminex assay and are depicted as pg per 100 mg colon tissue. Only three of the study groups were included in the analysis (12p40-mAb, rat-IgG2a, and SCID control mice).

**Table 2:** Changes in taxa abundance between active colitis (rat-IgG2a and NaCl) and nonactive disease (12p40-mAb and healthy) determined by ANOVA.

Phylum	Class	Order	Family	Genus	Relative distribution		Significance	
					Active colitis	Nonactive disease	*p* value	*q-*value^A^
Bacteroidetes	Bacteroidia	Bacteroidales	Bacteroidaceae	*Bacteroides*	37.19%	11.32%	0.002	0.060
Bacteroidetes	Bacteroidia	Bacteroidales	[Paraprevotellaceae]	Unclassified	0.14%	0.05%	0.004	0.072
Firmicutes	Clostridia	Clostridiales	Ruminococcaceae	*Ruminococcus*	0.47%	3.89%	0.004	0.054
Firmicutes	Clostridia	Clostridiales	Unclassified	Unclassified	13.22%	26.45%	0.010	0.100
Firmicutes	Clostridia	Clostridiales	Ruminococcaceae	*Oscillospira*	2.20%	4.21%	0.029	0.225
Bacteroidetes	Bacteroidia	Bacteroidales	Porphyromonadaceae	*Parabacteroides*	0.68%	0.15%	0.044	0.288
TM7	TM7-3	CW040	F16	Unclassified	0.01%	0.02%	0.045	0.249

^A^ *q*-values were determined with the False Discovery Rate correction method.

## Abbreviations

IBD: Inflammatory bowel disease
UC: Ulcerative colitis
CD: Crohn's disease
12p40-mAb: Rat anti-mouse IL-12/23p40 monoclonal antibody
IHC: Immunohistochemical
DAI: Disease activity index
MEICS: Murine endoscopy index of colitis severity
GM: Gut microbiota
OTU: Operational taxonomic unit
SCID: Severe combined immunodeficiency
AdTr-colitis: Adoptive transfer colitis.
