# Supplementary material for: Treatment with a Monoclonal Anti-IL-12p40 Antibody Induces Substantial Gut Microbiota Changes in an Experimental Colitis Model
Source: Gastroenterol Res Pract. 2016 Jan 6;2016:4953120. doi: 10.1155/2016/4953120 (PMC4736578; doi:10.1155/2016/4953120)
Supplement: Supplementary file 1 — Supplemental Material includes a Supplemental Figure and 4 Supplemental Tables. The Supplemental Figure contains DGGE profiles of 16S rRNA gene-amplicon of fecal, colon and cecum samples. The Supplemental Tables contain Murine endoscopic index of colitis severity (Supplemental Table 1), criteria for assignment of histopathology damage (Supplemental Table 2), bacterial richness as the number of OTU-species observed in colonic content (Supplemental Table 3) and r-values for significant correlations of immunological and host parameters with GM members (Supplemental Table 4). [file 4953120.f1.pdf]

Supplementary Data

Supplemental Table 1. Murine endoscopic index of colitis severity (MEICS)

Descriptor	0	1	2	3	Total
Thickening of the colon	Transparent	Moderate	Marked	Non-transparent	0-3
Changes of the vascular pattern	Normal	Moderate	Marked	Bleeding	0-3
Fibrin visible	None	Little	Marked	Extreme	0-3
Granularity of the mucosal surface	None	Moderate	Marked	Extreme	0-3
Stool consistency	Normal + solid	Still shaped	Unshaped	Spread	0-3
					Overall: 0-15

Supplemental Table 2. Histopathology score

Horizontal sections of colon representing oral and aboral segments are scored semi-quantitatively in a blinded fashion: No (0), mild (1), moderate (2) or severe (3) based on lesions, hyperplasia, crypt distortion and area involved (total score 0-12 per segment scored, i.e. total score 0-24 for oral and aboral colon). Scoring system adapted from Kjellev et al. [9]

**Score/Grade**	**No = 0**	**Mild = 1**	**Moderate = 2**	**Severe = 3**	**Total**
Severity of lesions	None	Lesions were small, focal, or widely separated multifocal areas of inflammation limited to the lamina propria	Lesions were either multifocal or represented by locally extensive areas of inflammation extending into the submucosa.	Lesions with extensive diffuse infiltration with inflammatory cells involving tunica mucosa, submucosa and potentially muscularis.	0-3
Hyperplasia	None	Hyperplasia consisted of morphologically normal lining epithelium that was up to one times thicker (length of crypts) than adjacent or control mucosa	Hyperplasia was characterized by the lining epithelium being above one to two times normal thickness, cells were hyperchromatic with mitotic figures.	Hyperplastic regions exhibited markedly thickened epithelium (two or more times normal), marked hyperchromasia of cells, few to no goblet cells, a high mitotic index of cells within the crypts.	0-3
Crypt distortion	None	Crypt damage consisted of damage of basal 1/3	Crypt damage consisted of damage of basal 2/3 with focal crypt loss and intact surface epithelium.	Crypt damage consisted of multifocal crypt loss with intact surface epithelium or focal crypt loss with no surface epithelium (erosion/ulceration).	0-3
Surface area involved by disease process	0-10%	>10%–40%	>40%–70%	>70%	0-3
Oral segment (0-12) + Aboral segment (0-12)					Overall: 0-24

Supplemental Table 3. Number of observed OTU-species estimated in the colonic content of non- and active colitis mice.

Number of Observed OTU-Species[A]				
Group 1		Group 2		*p*- value[B]
Healthy	498 ± 20	12p40-mAb	457 ± 76	> 0.05
Healthy	498 ± 20	NaCl	443 ± 64	> 0.05
12p40-mAb	457 ± 76	IgG2a	435 ± 111	> 0.05
IgG2a	435 ± 111	NaCl	443 ± 64	> 0.05
12p40-mAb	457 ± 76	NaCl	443 ± 64	> 0.05
Healthy	498 ± 20	IgG2a	435 ± 111	> 0.05

[A] Number of observed OTU-species was determined using 13,000 reads per sample.

[B] *p*-values were determined through a non-parametric two sample *t*-test.

Supplemental Table 4. Matrix containing the *r*- values for every significant correlation found using Pearson's correlation analysis of immunological and host parameters with microbiota members of the colonic content

IL_9	IL_10	IL_13	IL_2	IP_10	KC	TNF-α	CD3_density	Colon_ratio	Histopathology	Weight_AUC	Disease_AUC	Calprotectin	Taxa				
-0.52		-0.51			0.70	0.66	0.51	0.36	0.41	-0.40	0.38		Bacteroidetes	Bacteroidia	Bacteroidales	[Paraprevotellaceae]	Unclassified
	0.53												Bacteroidetes	Bacteroidia	Bacteroidales	[Paraprevotellaceae]	[*Prevotella*]
-0.5		-0.49	-0.49		0.64	0.67	0.78	0.54	0.59	-0.54	0.57	0.42	Bacteroidetes	Bacteroidia	Bacteroidales	Bacteroidaceae	*Bacteroides*
	0.57	0.53											Bacteroidetes	Bacteroidia	Bacteroidales	Prevotellaceae	*Prevotella*
				0.57		0.5	0.36		0.44				Bacteroidetes	Bacteroidia	Bacteroidales	Porphyromonadaceae	*Parabacteroides*
0.55	0.49	0.5											Bacteroidetes	Bacteroidia	Bacteroidales	Rikenellaceae	Unclassified
0.49	0.59	0.55	0.53				-0.43	-0.38			-0.45		Bacteroidetes	Bacteroidia	Bacteroidales	S24-7	Unclassified
					-0.51	-0.57							Firmicutes	Bacilli	Lactobacillales	Stretococcaceae	*Lactococcus*
							-0.44		-0.40			-0.41	Firmicutes	Clostridia	Clostridiales	Unclassified	Unclassified
				0.57	0.67	0.60			0.40			0.46	Firmicutes	Clostridia	Clostridiales	Clostridiaceae	Unclassified
							-0.42						Firmicutes	Clostridia	Clostridiales	Lachnosiraceae	Unclassified
					0.68	0.53							Firmicutes	Clostridia	Clostridiales	Lachnosiraceae	*Corococcus*
							-0.38		-0.39				Firmicutes	Clostridia	Clostridiales	Ruminococcaceae	*Oscillospira*
							-0.35			0.43			Firmicutes	Clostridia	Clostridiales	Ruminococcaceae	*Ruminococcus*
				0.56	0.54	0.54	0.44		0.39				Firmicutes	Erysielotrichi	Erysielotrichales	Erysielotrichaceae	Unclassified
											0.40		Proteobacteria	Alphaproteobacteria	RF32	Unclassified	Unclassified
					0.59	0.64	0.65	0.52	0.56	-0.52	0.63	0.38	Proteobacteria	Betaproteobacteria	Burkholderiales	Alcaligenaceae	*Sutterella*
												0.43	Unassigned				

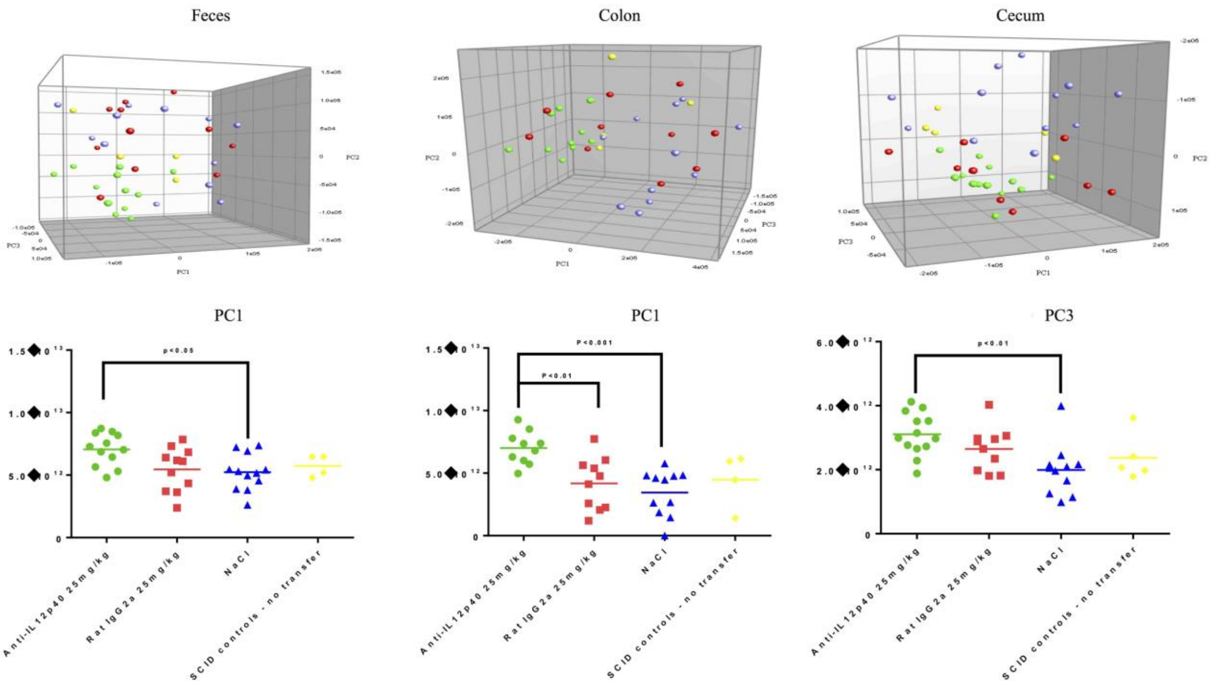

Supplemental Figure 1. Cluster analysis based on DGGE profiles of 16S rRNA gene-amplicon in feces, colon and cecum (Upper panels). Principle Coordinates (PC1 and PC3) used for comparison of treatment groups by Kruskal Wallis one-way ANOVA, followed by all-pairwise-multiple-comparison Dunn´s test (Lower panels).
